# Egg-yolk- and a liposome-based extenders: refrigeration time and effects on ram semen quality

**DOI:** 10.1590/1984-3143-AR2024-0052

**Published:** 2025-05-09

**Authors:** Rogério Araújo de Almeida, Luan Sitó da Silva, Luiz Gustavo Ferreira de Lima, Flávio Augusto Lourencetti, Camila Freitas-Dell´Aqua, João Carlos Pinheiro Ferreira, Eunice Oba

**Affiliations:** 1 Departamento de Cirurgia Veterinária e Reprodução Animal, Faculdade de Medicina Veterinária e Zootecnia, Universidade Estadual Paulista, Botucatu, SP, Brasil

**Keywords:** egg yolk, liposomes, cryoprotectant, freezing

## Abstract

Sperm cells require time to adjust to lower temperatures. While some studies have established stabilization periods ranging from two to four hours for ovine semen, the exploration of longer periods of stabilization remains limited. The objective of the study is to evaluate the 12-hour refrigeration curve during ovine semen cryopreservation and the impact of a diluent medium containing egg yolk and liposomes. Eight mixed-breed rams (½ Dorper and ½ Santa Inês) provided two ejaculates each, divided into two groups. Group A used a commercial egg yolk-based diluent (Botu-Bov® - Botupharma Ltda, Brazil), while Group B used a commercial liposome-based diluent (OptiXcell®, IMV Technologies, France). Semen was packaged in French straws, cooled, cryopreserved, and thawed for analysis. Group A exhibited superior values (P < 0.05) in progressive motility (MP), progressive linear velocity (VSL), straightness (STR), and linearity (LIN) post-thawing and after 3 hours at 37°C (TTR). Conversely, Group B showed higher (P < 0.05) values for path velocity (VAP), curvilinear velocity (VCL), lateral head displacement amplitude (ALH) post-thawing, and VAP, VSL, VCL, and ALH after TTR. Flow cytometry revealed Group A's higher (P > 0.05) plasma and acrosomal membrane integrity and membrane stability. However, Group B exhibited greater (P > 0.05) superoxide anion generation and lipid peroxidation, indicative of higher oxidative stress. In conclusion, the egg yolk-based diluent outperformed the diluent containing liposomes in sperm kinetic parameters evaluated by CASA, although liposomes showed increased oxidative stress, 12 hours of refrigeration at 5.0°C is an alternative viable for semen cryopreservation in sheep.

## Introduction

The development of AI in sheep has advanced alongside frozen semen biotechnologies; and cryopreservation allows semen to be stored indefinitely. Its usage has been restricted in the sheep industry likely due to low fertility (Maxwell and Watson, 1996) and is influenced by several factors, mainly due to the low post-thaw motility of the semen used (Palacios and Abecia, 2015). Frozen semen involves much higher selection pressure, resulting in a better genetic impact on the herd (Bicudo et al., 2005). Approximately half of the spermatozoa undergo cryodamage during cryopreservation (Maia, 2015). Thus, ensuring the availability of high-quality frozen or cooled semen, could enhance the use of cryopreserved ram semen.

Ram semen freezing entails collection, evaluation, extension, centrifugation or not, loading and sealing straws, cooling and stabilization curves, freezing curve, and then plunging into liquid nitrogen. The collection can be performed with electroejaculation or artificial vagina, with the latter preferred for longevity and welfare​ (Barbas et al., 2023; Prieto et al., 2014). Semen evaluation is mandatory for proper dilution before cryopreservation; and extension can be performed with extenders containing milk and milk-base proteins, egg-yolk, and liposomes, the latter being egg-yolk predominant​ (Argudo et al., 2019; Brasil et al., 2016). Centrifugation is an optional step in sheep semen cryopreservation, but it is recommended in goats(Prieto et al., 2014) . French straws are the primary method used to freeze semen of domesticated mammals ​(Argudo et al., 2019; Ntemka et al., 2018). Cooling followed by stabilization is needed to allow cryoprotectant to penetrate and interact with sperm membranes, protecting sperm from cryodamage (Prieto et al., 2014). Cooling curves begin with an equilibrium temperature of ~5°C, and the equilibrium period varies between species and protocols, after which the freezing curve passes ~5°C and drops to -79°C (Muiño et al., 2007; Prieto et al., 2014). Once the equilibrium status is attained, the semen is plunged into liquid nitrogen at -196°C​ (Argudo et al., 2019). All these steps have the potential to affect semen cryopreservation, however, semen extenders and stabilization time appear to play major roles (Ntemka et al., 2018)​.

The semen cryopreservation extenders contain intracellular (penetrating; e.g., glycerol and amides) and extracellular (non-penetrating; e.g., liposomes, egg-yolk, milk and products, mono and dissacharides, salt) cryoprotectants, In addition, extenders have buffers (e.g., TRIS and TES) and antibiotics (e.g., gentamicin and penicillin) (Bicudo et al., 2005). Egg yolk phospholipids, such as phosphatidylcholine, provide protection to the sperm cell in the freezing-thawing process and can be derived from other sources (Dorado et al., 2007). Liposomes derived from egg yolk phospholipids have also been effective in protecting spermatozoa during cryopreservation in stallions (Pillet et al., 2012) and bulls (Röpke et al., 2011), while allowing for a standardized composition in extender. Commercial semen extenders containing egg-yolk and liposome are available for sheep but their effectiveness remains to be compared. However, egg yolk can potentially carry diseases, increase sperm agglutination, and induce pre-capacitation, all of which can influence semen fertility (Mehdipour et al., 2016; Najafi et al., 2017). Thus, the absence of biologically derived animal products in extenders is highly desirable.

In practice with ruminants, semen is collected in the field and transported to breeding centers for cryopreservation. After processing, cooling and stabilization are performed for 2 – 12h (Barbas and Mascarenhas, 2009; Hameed et al., 2024). Cooling curve is followed by stabilization to allow sperm cells to adapt to low temperatures and the different components of the extender(Leite et al., 2010). Under well-controlled conditions, ram semen is st for 2-4 hours before cryopreservation (Fiser et al., 1986; Mafolo et al., 2020). However, in private practice ram semen is typically stored for 12-hour before cryopreservation. Most of breeding centers follow this prolong stabilization time as the large majority of the semen shipped overnight at ~5^o^C for cooled use or cryopreserved in the morning. While, this may not be the most suitable stabilization time, most laboratories follow this practice to maximize their capacity.

The overall objective of this study was to compare ram semen cryopreserved with an egg-yolk- and a liposome-based extenders. The specific objectives were to assess: (i) sperm motility parameters, (ii) plasma membrane and acrosome integrity, (iii) membrane instability and lipid peroxidation. The hypothesis is the liposome based-extender cryopreserves ram semen better than the egg-yolk-based extender after a 12-hour cooling period.

## Methods

The project was conducted following ethical guidelines recommended by the Brazilian College of Animal Experimentation (COBEA) and approved by the Ethics Committee on Animal Use (Protocol number CEUA 0467/2023).

The study was performed at the Laboratory of Biotechnology Applied to the Reproduction of Small Ruminants of the Department of Veterinary Surgery and Animal Reproduction, FMVZ – UNESP, Botucatu, São Paulo, Brazil; South latitude 22º53'09”; West longitude 48º26'42”, with an altitude of 804 meters. The study was conducted during the physiological breeding season of the Southern hemisphere (June 2023) for small ruminants.

### Animals

Eight mixed-breed sheep, ½ Dorper and ½ Santa Inês, approximately 18 months old, were employed in this study. These sheep were obtained from the Faculty of Veterinary Medicine and Animal Science of Botucatu, UNESP, Botucatu, SP. They were deemed reproductively fit based on established standards (Colégio Brasileiro De Reprodução Animal, 2013) and were clinically healthy. The animals were individually housed in free-exhaustion bays, sheltered from the sun, and received a diet comprising corn silage and maintenance feed twice a day (8 AM and 4 PM). Clean water was available ad libitum from water troughs.

### Semen collection and processing

The animals were already accustomed to semen collection and were on a weekly collection routine. Semen collection was carried out using an artificial vagina (short model preheated to 42°C), with a sheep serving as a model. Two ejaculates were collected from each animal, totaling sixteen samples. After collection and pre-analysis, the ejaculate was divided into two groups: Group A (egg yolk-based diluent; Botu-Bov® - Botupharma Ltda, Botucatu, São Paulo, Brazil) and Group B (liposome-based diluent; OptiXcell® 2 (⅓) and ultrapure water (⅔) - IMV Technologies, Campinas, São Paulo, Brazil).

### Semen freezing

With a minimum concentration of 200x106 sperm per mL and initial total motility of at least 75%, the semen was distributed in 0.25 ml French straws, 4 straws/group/animal were packaged. Cooling was performed in a Minitub® refrigerator, 518°C (Minitub do Brasil Ltda, Porto Alegre, Brazil), where the straws were placed horizontally in the support and remained refrigerated at 5.0°C for 12 h. Subsequently, the support was transferred to a 44-liter box containing liquid nitrogen. Then, the straws were positioned three centimeters above the liquid, only in nitrogen vapor, in the horizontal position, for 20 min at -120.0°C. They were then submerged in nitrogen at -196.0°C (Crespilho et al., 2012), placed in supports and stored in cryogenic cylinders for later analyses.

For sperm concentration, 10 μL of the semen sample was diluted in 4 mL of distilled water at a dilution rate of 1:400 for sperm concentration assessment using a Neubauer chamber under an optical microscope at 200x magnification. A second aliquot of 10 μL was used for sperm morphological evaluation with the assistance of a phase-contrast microscope, counting 200 cells (Colégio Brasileiro De Reprodução Animal, 2013).

### Computerized sperm evaluation post-thaw

For computerized evaluation of sperm kinetics, a semen sample was thawed in a water bath at 37°C for at least 30 seconds, an aliquot of 10 µL of semen and 1,000 µL of diluent was placed in the Mackler chamber and subjected to computerized assessment of sperm quality (Hamilton Thorn Research – IVOS® 12, Beverly, Massachusetts, USA), previously adjusted for ovine species seminal evaluation. In each sample, five randomly chosen fields were evaluated, with a minimum of 200 spermatozoa per field, totaling 1000 cells. The following parameters were assessed: total sperm motility (TM, %); progressive sperm motility (PM, %); average trajectory speed (VAP, mm/s); curvilinear speed (VCL, mm/s); linear progressive speed (VSL, mm/s); lateral head displacement amplitude (ALH, mm); tail beat frequency (BCF); straightness (STR); linearity (LIN, %); and the percentage of spermatozoa with rapid movement (RAP).

### Sperm evaluation by flow cytometry

For flow cytometry sperm evaluation, another semen sample was thawed in a water bath at 37°C for at least 30 seconds, the BD LSR Fortessa equipment (Becton Dickinson, Mountain View, CA, USA) was used, equipped with excitation lasers: blue 488-nm, 100 mW, and emission filters 530/30 nm (FITC, YOPRO, and CM-H2DCFDA) and 695/40 nm (Propidium iodide and MitoSOX Red); red 640-nm, 40 mW, with a 660/20 nm filter (MitoStatus Red); and violet 405-nm, 100 mW, with a 450/50 nm filter (Hoechst). At least 10,000 cells per sample were analyzed, and the data were evaluated using BD FACSDiva™ software v 6.1.

Samples were diluted in TALP-PVA: 100 mM NaCl, 3.1 mM KCl, 25.0 mM NaHCO3, 0.3 mM NaH2PO4, 21.6 mM DL-sodium lactate 60%, 2.0 mM CaCl2, 0.4 mM MgCl2, 10.0 mM Hepes-free acid, 1.0 mM sodium pyruvate, 1.0 mg/mL polyvinyl alcohol-PVA, and 25 µg/mL gentamicin) at a concentration of 5 x 10^6 spermatozoa/ml, supplemented with Hoechst 33342 (7 µM; 145333, Sigma) to eliminate debris (Parrish et al., 1988).

For the assessment of plasma and acrosomal membrane integrity, the combination of propidium iodide (PI; P4170, Sigma) and FITC-PSA (Pisum sativum agglutinin conjugated to fluorescein isothiocyanate; L0770, Sigma) (Freitas-Dell’Aqua et al., 2012). Thus, in a 200 µL sample of diluted semen, 1.5 µM of PI and 2 ng of FITC-PSA were added, and the samples were incubated for 15 min at 37°C, protected from light.

For the evaluation of plasma membrane destabilization, mitochondrial membrane potential, and superoxide (O2•-) production in the mitochondrial matrix, the combination of YOPRO (YP; labeling for cells with destabilized plasma membrane; Y3603, Life Technologies), MitoStatus Red (MST; mitochondrial potential; 564697, BD Pharmigen), and MitoSOX™ Red (MSR; superoxide anion generation in the mitochondrial matrix, M36008, Life Technologies) (Freitas-Dell’Aqua et al., 2018). Thus, in a 500 µL sample of diluted semen, 25 nM YP, 20 µM MST, and 2 µM MSR were added, followed by incubation at 37°C for 20 minutes.

For lipid peroxidation, the thawed samples were washed by centrifugation (300g/5min) to remove the diluent medium, reducing the influence of lipids present in the diluents on the analysis results. After this wash, the protocol (Guasti et al., 2012) was used, using the C11-BODYPY probe (D-3861; Molecular Probes). Thus, in 500 μL of diluted semen in TALP-PVA, 5 μM of C11-BODIPY581/591 was added, followed by incubation for 30 minutes at 37°C. After incubation, two consecutive washes were performed by centrifugation at 300g for 5 minutes, with TALP-PVA, and the pellet was resuspended in 300 μL of TALP-PVA. This assessment will be performed at 5 and 120 minutes post-thawing with samples kept at 37°C.

### Data analysis

The sample size was calculated using SAS 3.81 (Enterprise Edition) software, taking into account the means and standard deviation of progressive motility (which had the highest variance) from a previously conducted study (means = 37.1; 30.3; 31.4; 31.0; Standard Deviation = 4.1). This study indicated a sample of 7.28 individuals per group to achieve a test power > 0.8 with alpha = 0.5.

Variables showing a normal distribution were represented by mean and standard deviation, and comparison of means was conducted using a paired t-test. Non-normally distributed variables were logarithm-transformed. The significance level was set at α = 0.05. All statistical analyses were performed using SAS 3.81 (Enterprise Edition) software.

## Results

**Figure 1:**
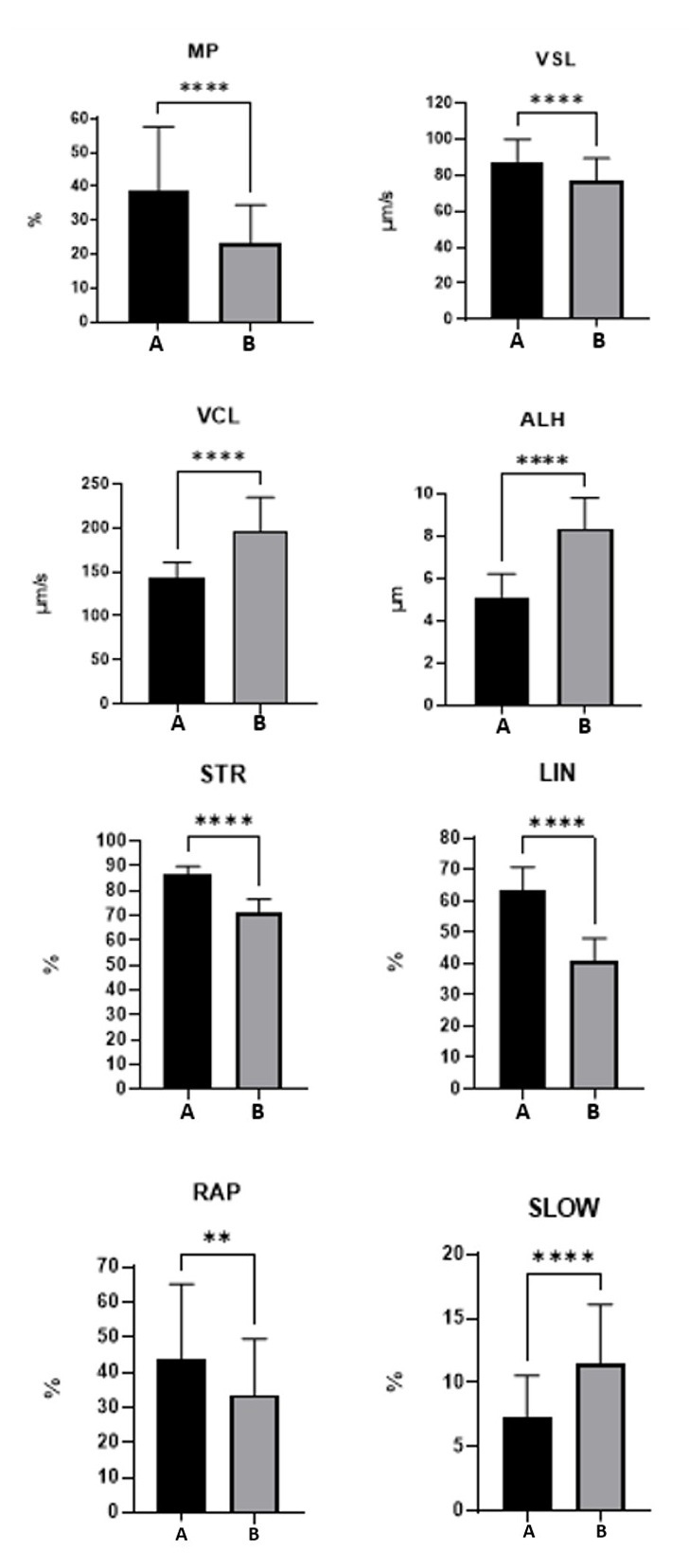
Column charts generated by the Paired T-Test to determine statistical differences between variables in frozen ovine semen with egg yolk-based medium (Group A, black) or ovine semen frozen with liposome-based medium (Group B, gray) and significant difference (P < 0.0001). Legend: Progressive Motility (MP), Progressive Linear Velocity (VSL), Curvilinear Velocity (VCL), Lateral Head Displacement Amplitude (ALH), Straightness (STR), Linearity (LIN), Percentage of Sperm with Rapid Movement (RAP), and Percentage of Sperm with Slow Movement (SLOW).

The evaluation immediately after thawing of semen with egg yolk-based diluents (Group A) or liposomes-based diluents (Group B) revealed that the mean values of Group A had higher values (<0.0001) for progressive motility (MP), curvilinear velocity (VCL), progressive linear velocity (VSL), lateral head displacement amplitude (ALH), linearity (LIN), straightness (STR) and percentage of sperm with slow movement (SLOW). On the other hand, Group B showed higher values (<0.0001) compared to Group A for curvilinear velocity (VCL), and for lateral head displacement amplitude (ALH) (Table 1). Meanwhile, parameters of total motility (MT), mean average path velocity (VAP), and tail beat frequency (BCF) showed no statistical difference (Figure 1).

**Table 1:** Mean and standard deviation of parameters obtained from computerized evaluation of sperm kinetics, performed using the Hamilton Thorn Research – IVOS ® 12 equipment, from ovine semen immediately after thawing. Data from 8 animals, 2 ejaculates each (n=16), divided into two diluent groups, Group A (egg yolk-based), Group B (liposome-based).

**Variable**	**Group A**	**Group B**	**P Value**
MT %	58.1±23.8	53.7±22.0	NS
MP %	38.7±18.9	23.1±11.4	<0.0001
VAP mm/s	98.4±12.8	104.5±17.7	NS
VSL mm/s	87.5±12.2	76.9±12.3	<0.0001
VCL mm/s	142.8±18.2	196.6±37.8	<0.0001
ALH mm	5.1±1.1	8.3±1.4	<0.0001
BCF hz	36±3.2	33.9±3.8	NS
STR %	86.9±2.9	71.3±5.29	<0.0001
LIN %	63.4±7.3	40.9±7.1	<0.0001
RAP %	43.8±21.3	33.5±16	<0.05
SLOW %	7.3±3.1	11.5±4.6	<0.0001

Legend: Not significant (NS); Total Sperm Motility (MT); Progressive Sperm Motility (MP); Average Path Velocity (VAP); Curvilinear Velocity (VCL); Progressive Linear Velocity (VSL); Lateral Head Displacement Amplitude (ALH); Tail Beat Frequency (BCF); Straightness (STR); Linearity (LIN); Percentage of Sperm with Rapid Movement (RAP); Percentage of Sperm with Slow Movement (SLOW).

The data after the 3-hour thermoresistance test at 37°C are presented in Table 2. It is observed that group A still showed higher values (P>0.05) for MP and LIN compared to group B. On the other hand, group B demonstrated higher values (P<0.05) compared to A for the parameters of VAP, VSL, VCL and ALH. For the values obtained by the parameters of MT, BCF, and STR, there was no significant difference.

**Table 2:** Mean and standard deviation of parameters obtained from computerized analysis of sperm kinetics, performed using the Hamilton Thorn Research – IVOS® 12 equipment, of thawed ovine semen subjected to a 3-hour thermoresistance test at 37°C. Data from 8 animals, 2 ejaculates each (n=16), divided into two diluent groups, Group A (egg yolk-based), and Group B (liposome-based).

**Variable**	**Group A**	**Group B**	**P Value**
MT %	44±26	45.1±19.1	NS
MP %	29±18,9	21.9±10.6	0.03
VAP mm/s	80.9±13.5	105.3±16.4	<0.0001
VSL mm/s	71.2±11.7	78.4±11	0.03
VCL mm/s	125.4±23.6	192.9±28.9	<0.0001
ALH mm	4.9±1	8.2±1	<0.0001
BCF hz	33.7±3,9	33±3.6	NS
STR %	82.2±19.8	73.9±3.4	NS
LIN %	60.7±6.3	42.6±3.2	<0.0001
RAP %	32.5±22.1	31.2±16.4	NS
SLOW %	5.5±4.5	8.6±3	<0.05

Legend: Not significant (NS); Total sperm motility (MT); Progressive sperm motility (MP); Average path velocity (VAP); Curvilinear velocity (VCL); Progressive linear velocity (VSL); Amplitude of lateral head displacement (ALH); Beat cross frequency (BCF); Straightness (STR); Linearity (LIN); Percentage of sperm with rapid movement (RAP); Percentage of sperm with slow movement (SLOW).

In the flow cytometry analysis (Table 3), immediately after thawing, group A showed higher results (P>0.05) for damaged plasma membrane and intact acrosome (MPLAI: 37.1±4.4 vs. 26.9±3.3); intact plasma membrane and acrosome (MPAI: 28.3±3.5 vs. 18.1±2.4), and membrane stability (38.8±3.3 vs. 20.5±2.8) compared to group B. For the parameters of intact plasma membrane and damaged acrosome (MPAL), the percentage of cells with high mitochondrial potential (HPM), and intensity of mitochondrial potential in stable cells (MS), there was no significance. Furthermore, in the flow cytometry evaluation, oxidative stress assessed by the generation of superoxide anion and lipid peroxidation was higher in group B (P>0.05) than in group A (Table 4).

**Table 3:** Mean and standard deviation of parameters obtained from flow cytometry evaluation for sperm membrane, performed by the LSRFortessa equipment, of ovine semen immediately after thawing. Data from 8 animals, 2 ejaculates each (n=16) divided into two dilution media, group A (egg yolk-based), group B (liposome-based).

**Variable**	**Group A**	**Group B**	**P Value**
MPLAI %	37.1±4.4ª	26.9±3.3^b^	0.0064
MPAL %	31.2±3.7^b^	52.7±3.1ª	< 0.0001
MPAI %	28.3±3.5ª	18.1±2.4^b^	0.0005
MPIAL %	3.3±0.8	2.0±0.4	NS
MS %	38.8±3.3ª	20.5±2.8^b^	< 0.0001

Legend: MPLAI = Damaged plasma membrane and intact acrosome; MPAL = Damaged plasma membrane and acrosome; MPAI = Intact plasma membrane and acrosome; MPIAL = Intact plasma membrane and damaged acrosome; MS: Membrane stability.

**Table 4:** Mean and standard deviation of parameters obtained from flow cytometry evaluation for lipid peroxidation, conducted by the LSRFortessa equipment, of ovine semen immediately after thawing. Data from 8 animals, 2 ejaculates each (n=16) divided into two dilution media, group A (egg yolk-based), group B (liposome-based).

**Variable**	**Group A**	**Group B**	**P Value**
HPM %	58.9±3.9	55.5±3.4	NS
PMSC UA	14,860±1476	18,053±1251	NS
O2 (SO)	61.9±3.1ª	76.7±2.7ª	< 0.0001
SO TOTAL	1,747±274^b^	2782±2ª	0.0005
PER. L	52±2.6^b^	65.3±1.3ª	0.0006
PER. L TOTAL	1,223±70.2^b^	1514±41.2ª	0.0006

Legend: HPM: high mitochondrial potential; PMSC: Stable cells with high mitochondrial potential; SO: superoxide anion; SO TOTAL: total cells with oxygen; O2 HIGH: cells with high oxygen rate; PER. L: lipid peroxidation; PER.L TOTAL: total lipid peroxidation; P. HIGH: high lipid peroxidation.

## Discussion

The sperm kinetics results show that group A presented more rectilinear movement after thawing, as demonstrated by MP, VSL, VCL, STR, and LIN values, as well as after heat stress with higher MP and LIN. Velocity values are determined by the distance covered in a period of time, while LIN and STR values are calculated based on the ratio of velocity parameters (Amann and Katz, 2004). Compared to group B, group A had a higher percentage of cells with a linear index, which represents the ratio between VSL and VCL. As spermatozoa deviate from a straight path, their linearity decreases (Mortimer, 2000).

In this study, the liposome-based extender showed lower post-thaw sperm kinetics compared to the egg yolk-based extender after 12 hours of refrigeration. Lower sperm viability and motility rates were reported in Bapedi sheep using a liposome-based extender without egg yolk, though in that case, a cooling curve of only two hours was applied (Mafolo et al., 2020). Despite varying liposome concentrations, no improvements were seen in total motility, progressive motility, or fast sperm percentages. Similarly, reduced motility values were observed in stallion semen extended with liposomes (E80), which showed significantly lower VAP, PM, and RAP values compared to egg yolk extenders (Pillet et al., 2012).

The 12-hour stabilization curve in this study indicated that egg yolk extenders (Group A) better preserved plasma membrane integrity and stability compared to liposome-based extenders (Group B). This finding contradicts a study on Dorper sheep, which reported higher sperm viability and membrane integrity with liposomes under a shorter, 4-hour refrigeration protocol (Luna-Orozco et al., 2019). The mechanisms by which liposomes stabilize cells during freezing remain poorly understood (Fleisch et al., 2017). One hypothesis is that liposomes modify the sperm membrane by exchanging lipids and cholesterol at lower temperatures (Sullivan and Saez, 2013). Cholesterol exchange with the sperm lipid bilayer occurs relatively quickly (Bruckdorfer et al., 1969), but lipid transfer is slower (Phillips and Lardy, 1940), suggesting that a longer equilibration time may be needed to enhance sperm kinetic values in ovine semen (Muiño et al., 2007). A study reported that extending the equilibration time to 24-72 hours with OPTIXcell® or Triladyl-egg yolk® extenders improved sperm motility and viability in bull semen, although no difference was found in field fertility between the 4-hour and 72-hour curves (Fleisch et al., 2017).

In this study, the 12-hour cooling period at 5°C was in accordance with the laboratory’s freezing routine, and the incubation time for the commercial liposome-based extender (Group B) followed the manufacturer’s recommendations. Studies indicate that longer stabilization periods, such as 24 hours, before freezing bovine semen result in improved membrane integrity (Röpke et al., 2011). The 12-hour equilibration at 5°C appeared sufficient for egg yolk-based extenders but may have been too short for liposome-based extenders. Extending equilibration times to 24-72 hours might allow more time for lipid exchange and membrane stabilization (Fleisch et al., 2017). Additionally, longer equilibration may improve membrane fluidity and protection against oxidative stress, which is crucial for maintaining sperm function post-thaw (Röpke et al., 2011).

The specific influence of liposomes on cellular membranes is determined by the physical and chemical properties of the lipids involved, such as the length of acyl chains, the number of double bonds, and the type and charge of the lipid headgroup (Batista et al., 2007; Frézard et al., 2005; Lasic, 1998). The liposome-based extender (Group B) resulted in lower progressive motility and membrane integrity, likely due to the slower interaction between liposomes and the sperm membrane during the 12-hour equilibrium period. Liposomes, while offering a contaminant-free alternative to egg yolk, may not engage as effectively with the sperm membrane over shorter equilibration times (Röpke et al., 2011). Made of synthetic phospholipids like phosphatidylcholine, liposomes are designed to mimic natural membranes; however, the slow exchange of lipids can result in reduced protection against cryoinjury, which is reflected in the higher levels of membrane damage and oxidative stress observed in Group B (Aitken and Baker, 2004; Chelucci et al., 2015; Ricker et al., 2006).

Liposomes composed of phosphatidylserine and cholesterol (PSCH) have been shown to improve sperm recovery post-thaw in stallions but failed to induce an acrosomal reaction in sperm treated with dilauroylphosphatidylcholine (PC12) (Wilhelm et al., 1996). This suggests that phosphatidylserine and cholesterol (PSCH) could enhance flow cytometry results in ovine semen.

The higher oxidative stress in Group B, identified through flow cytometry, may be due to an imbalance between the production of reactive oxygen species (ROS) and the sperm's antioxidant capacity. ROS can cause lipid peroxidation and DNA damage (Devi et al., 2000). Ovine spermatozoa have high levels of polyunsaturated fatty acids in their plasma membranes, making them especially vulnerable to lipid peroxidation (Aitken and Baker, 2004). The increased oxidative stress in Group B likely contributed to reduced sperm motility and membrane integrity, this could be due to the damage that reactive oxygen species (ROS) produced by oxidative stress cause to the sperm membrane

Egg yolk-based extenders are traditionally preferred due to the protective properties of low-density lipoproteins (LDL) found in egg yolk. These LDLs interact with the sperm membrane, reducing cryoinjury during the freeze-thaw process by stabilizing membrane fluidity and preventing damage. LDLs protect the integrity of membrane phospholipids during cryopreservation (Wang et al., 2014).

In this study, semen was diluted directly into the freezing extender without removing the ovine seminal plasma. It's known that seminal plasma components, including specific proteins and epididymosomes, affect cryostability in cattle and stallions (Manjunath et al., 2002; Röpke et al., 2011; Scolari et al., 2010). This raises the hypothesis that excluding ovine seminal plasma could improve membrane integrity and sperm kinetics during cryopreservation.

In stallions, post-thaw motility values were significantly higher (P < 0.0001) in semen extended with egg yolk compared to a liposome-containing group, though there was no significant difference in fertility after insemination (P = 0.23) (Pillet et al., 2012). With this, we can hypothesize that even with lower values ​​than egg yolk, semen diluted with liposomes may be an alternative for use in sheep reproduction systems. Future research should aim to optimize the interaction between liposome-based extenders and sperm membranes, possibly by extending equilibration times or incorporating additional protective agents to enhance cryoprotective effects.

## Conclusion

For sperm kinetics, motility, and plasma membrane integrity, the egg yolk-based extender proves superior to liposomes for ram semen cryopreservation, however, the cooling curve at 5°C for 12 hours is a viable alternative for cryopreservation of sheep semen.

## Data Availability

Research data is only available upon request.
